# Cognitive potency and safety of tDCS treatment for major depressive disorder: a systematic review and meta-analysis

**DOI:** 10.3389/fnhum.2024.1458295

**Published:** 2024-09-16

**Authors:** Junjie Wang, Xinru Yao, Yuqi Ji, Hong Li

**Affiliations:** ^1^School of Psychology, Capital Normal University, Beijing, China; ^2^Beijing Key Laboratory of Learning and Cognition, School of Psychology, Capital Normal University, Beijing, China; ^3^Department of Psychology, University of Tuebingen, Tübingen, Germany; ^4^Department of Psychiatry, The First Hospital of Shanxi Medical University, Taiyuan, China; ^5^Shanxi Key Laboratory of Artificial Intelligence Assisted Diagnosis and Treatment for Mental Disorder, The First Hospital of Shanxi Medical University, Taiyuan, China

**Keywords:** transcranial direct current stimulation, depression, treatment, cognition, meta-analysis

## Abstract

**Background:**

The benefits of transcranial direct current stimulation (tDCS) for patients with major depression disorders are well-established, however, there is a notable research gap concerning its comprehensive effects on both depressive symptoms and cognitive functions. Existing research is inconclusive regarding the cognitive enhancement effects of tDCS specifically in MDD patients. The present study aims to fill this knowledge gap by scrutinizing the most updated evidence on the effectiveness of tDCS in anti-depressive treatment and its influence on cognitive function.

**Methods:**

A systematic review was performed from the first date available in PubMed, EMBASE, Cochrane Library, and additional sources published in English from 1 January 2001 to 31 May 2023. We examined cognitive outcomes from randomized, sham-controlled trials of tDCS treatment for major depression. The evaluation process strictly followed the Cochrane bias risk assessment tool into the literature, and meta-analysis was performed according to the Cochrane System Reviewer's Manual.

**Results:**

In this quantitative synthesis, we incorporated data from a total of 371 patients across 12 studies. Results showed significant benefits following active tDCS compared to sham for the antidepressant effect [SMD: −0.77 (−1.44, −0.11)]. Furthermore, active relative to sham tDCS treatment was associated with increased performance gains on a measure of verbal memory [SMD: 0.30 (−0.02, 0.62)]. These results did not indicate any cognitive enhancement after active tDCS relative to sham for global cognitive function, whereas there was a noticeable trend toward statistical significance specifically in the effect of verbal memory.

**Conclusions:**

Our study offers crucial evidence-based medical support for tDCS in antidepressant and dimension-specific cognitive benefits. Further well-designed, large-scale randomized sham-controlled trials are warranted to further validate these findings.

**Systematic Review Registration:**

https://inplasy.com/, identifier: INPLASY202360008.

## 1 Introduction

Depression, as a neuropsychiatric condition, frequently coexists with chronic pain, a multifaceted disorder characterized by a constellation of sensory, cognitive, and affective symptoms (Chopra and Arora, 2014). Indeed, clinical research has underscored the significant comorbidity between pain and depression, with a notable study revealing a prevalence of 30% comorbidity between the two conditions, wherein each condition reciprocally exacerbates the other (Kroenke et al., 2011; Miller and Cano, 2009). Furthermore, prior research has revealed that patients enduring cognitive impairment often encounter unfavorable clinical outcomes (Hale et al., 2020; Ismail et al., 2017). Within this population, major depression disorder (MDD) is linked to notable decrements in executive function, working memory, and attention (Keefe et al., 2014; Wagner et al., 2012), potentially resulting in increased healthcare utilization and costs (Egede, 2007), as well as decreased adherence to medical treatments (DiMatteo et al., 2000). Thus, successfully treating depression could significantly enhance cognitive functions and improve prognosis in these patients. In recent years, psychiatric centers and health care services have increasingly emphasized the integration of physiotherapy for cognitive remediation, aiming to promote recovery and expand physical therapy for coping with mental illness (Douglas et al., 2022; Poppe et al., 2021).

Transcranial direct current stimulation (tDCS) has been explored as a physical intervention that may help alleviate depression symptoms and enhance cognitive functions for patients with MDD (Martin et al., 2018). The last decades saw an important growth of demand for this treatment, leading to a greater need for more efficient and safe methods of delivery. On the other hand, tDCS may be hampered by the therapeutic difficulties common to all forms of physical therapy, e.g., optimal parameters and treatment duration. Previously, tDCS involved applying small direct current to the brain through two electrodes placed on the scalp. In modern clinical trials for depression, tDCS is typically administered for 10–30 min per session, with a current intensity ranging from 1 to 2.5mA. This treatment is often administered once or twice daily over several weeks (Brunoni et al., 2016). Typically, these trials involve placing an anode or excitatory stimulus on an individual's left dorsolateral prefrontal cortex (DLPFC), while the cathode or reference electrode is positioned frontally on the contralateral side of the brain. Notably, the DLPFC is considered as a crucial hub within the “cognitive control” network that is often dysfunctional in depression (Williams, 2017). Current evidence suggests that tDCS possesses potent antidepressant properties. However, its effectiveness in enhancing cognitive function in patients with MDD remains inconclusive (Brunoni et al., 2017; Lefaucheur et al., 2017).

Cognitive dysfunction is a prominent symptom of MDD (Culpepper et al., 2017), manifesting as moderate impairments in executive function, memory, and attention in depressed patients (Rock et al., 2014). In patients with comorbid depression and cognitive dysfunction, previous randomized controlled trials (RCTs) have indicated that psychotherapy can be an effective treatment option (Miguel et al., 2023). Notably, some studies have observed cognitive benefits in patients with major depression following a series of tDCS sessions. However, a meta-analysis conducted by Martin et al. (2018) found no significant cognitive enhancement following active tDCS compared to sham for 12 cognitive outcomes across 478 MDD patients. While more clinical trials on cognitive functions in depressed patients have been published in recent years, potentially altering previous findings, the exact extent of tDCS effects on depression, cognitive-related outcomes (such as verbal memory and executive function), and adverse events (like pain and headache) remains uncertain. There is ongoing debate about which specific cognitive dimensions are most affected by tDCS. Additionally, current clinical research on tDCS often involves small sample sizes or even case reports, leading to inconsistencies in overall results. Finally, there is a scarcity of systematic reviews and meta-analyses assessing the effectiveness of tDCS in improving cognitive function in depression. Consequently, there is a pressing need for a comprehensive assessment of the evidence regarding tDCS's efficacy and its impact on cognitive function.

Thus, we aimed to determine whether a tDCS treatment course for major depression induces cognitive enhancement. We focused specifically on RCTs that evaluated cognitive functions in MDD patients receiving tDCS treatment. This targeted approach was taken given the increasing clinical importance of addressing cognitive impairments in depression and the need for evidence-based information on the cognitive effects of tDCS. However, it is acknowledged that this decision limited the sample scope and may have introduced bias by excluding studies that solely assessed the antidepressant efficacy of tDCS without considering cognitive outcomes. Despite this limitation, the aim was to provide a focused investigation into the potential cognitive benefits of tDCS for MDD patients. Current studies on tDCS's role in enhancing cognitive functions in depression yield conflicting results. This inconsistency underscores the pressing need for a comprehensive meta-analysis to systematically assess tDCS's therapeutic effectiveness. These analyses will clarify the overall impact of tDCS on cognitive functions in depressed patients, identify potential moderators of treatment efficacy, and guide future research and clinical applications.

In the current study, cognitive measures represented key cognitive domains including global cognitive function, verbal memory, executive function, and working memory. Through these efforts, we endeavor to deepen our understanding of the effectiveness of tDCS in anti-depressive treatment and its impact on cognitive function. Of note, we focused exclusively on MDD patients due to their significant cognitive impairments affecting daily life. Subthreshold depression patients have less severe symptoms and potentially lesser cognitive issues. To ensure consistency and statistical power, we included only MDD patients meeting diagnostic criteria. This understanding would serve as a solid foundation for further promoting the clinical application of tDCS.

## 2 Methods

### 2.1 Protocol registration

A systematic literature review and meta-analysis was conducted followed INPLASY (https://inplasy.com/) procedures and focused on the tDCS treatment for MDD patients with cognitive dysfunction. This review protocol was pre-registered in INPLASY (INPLASY202360008) and adhered to the Preferred Reporting Items for Systematic Reviews and Meta-analyses (PRISMA) reporting guideline (Moher et al., 2009).

### 2.2 Search strategy

We performed a systematic literature search in Pubmed, Embase, Web of Science, and Cochrane library, using both keywords and MeSH terms in the database ranged from 1 January 2008 to 31 May 2023. For additional references, we also searched the reference lists from the selected papers and other systematic meta reviews. The search strings for literature reviews included terms “depression,” “depressive disorder,” “dysthymi^*^,” “affective disorder,” “mood disorder,” “mood disorders,” “depression^*^,” “depressive^*^,” “transcranial direct current stimulation,” and “tDCS.” Two researchers reviewed and screened all records, and if there were disagreements, then they were resolved by consensus.

### 2.3 Eligibility criteria

For this systematic review and meta-analysis, we included: (1) subjects: patients with a disease diagnosis that met the Diagnostic and Statistical Manual of Mental Disorders (DSM) diagnostic criteria for depressive episodes to ensure homogeneity across studies; (2) age ≥ 18 years; (3) interventions: tDCS group and sham tDCS; (4) randomized sham-controlled trials (RCTs), which provide the highest level of evidence and minimize biases; (5) standardized neuropsychological test was performed at baseline and after treatment, and (6) peer-reviewed manuscripts in English.

Exclusion criteria: (1) missing full text or original data (e.g., meeting abstracts); (2) high-risk bias: studies were assessed using the Cochrane Risk of Bias Assessment Tool and excluded if four or more of them were high risk; (3) repeatedly published literature; (4) animal experiments, review literature, case studies were excluded to maintain the robustness and validity of our results; (5) unclear description of intervention methods, e.g., only including the tDCS group without a control group; and (6) lack of a sham-control group as well as neuropsychological test.

### 2.4 Data extraction

The retrieved documents were imported into EndnoteX9. Two researchers independently extracted data from qualified studies regarding author, publication year, sample size, average age, clinical characteristics, tDCS parameters, and neuropsychological performance. We extracted test score with standard deviation (SD), sample size, and *P*-values for effective size (ES) generation.

The primary outcomes included total scores on the depression scale and ratings across neuropsychological tests dimensions, such as global cognitive function, verbal memory, attention, executive function, and other cognitive dimensions. Secondary outcome refers to adverse events in this study.

### 2.5 Quality assessment

Study data were extracted by two independent reviewers, using consensus discussions for disagreements, and final data verifications conducted by the study statistician. The reviewers separately evaluated eligible studies using the Cochrane Collaboration Risk of Bias (RoB) tool: (a) random sequence generation; (b) allocation concealment; (c) blinding of participants and personnel; (d) blinding of outcome assessment; (e) Incomplete outcome data; (f) Selective reporting; (g) other bias, e.g., quality control of treatment procedures, adverse events (Higgins et al., 2011). The level of risk of bias is expressed as “low risk” and “high risk”, respectively, and “unclear” is used when the article has insufficient information.

### 2.6 Data analysis

Review Manager 5.3 software was used to assess the risk bias of included qualified studies. The effect size of heterogeneity of the studies was assessed by *I*^2^ statistic and *P*-values: *I*^2^ > 50% or *P* < 0.05 indicates high heterogeneity, and the random-effects model is used for meta-analysis; *I*^2^ ≤ 50% or *P* > 0.1, indicating that the research is homogeneous, then the fixed effects model is used. Meta-analysis was carried out according to the Cochrane System Reviewer's Manual. Observation indicators included in this study are continuous variables, since the scores of each test are continuous variables and the scale version used in each document is different, the standardized mean difference (SMD) is selected as the combined effect size.

## 3 Results

### 3.1 Study selection and study characteristics

**Figure 1:**
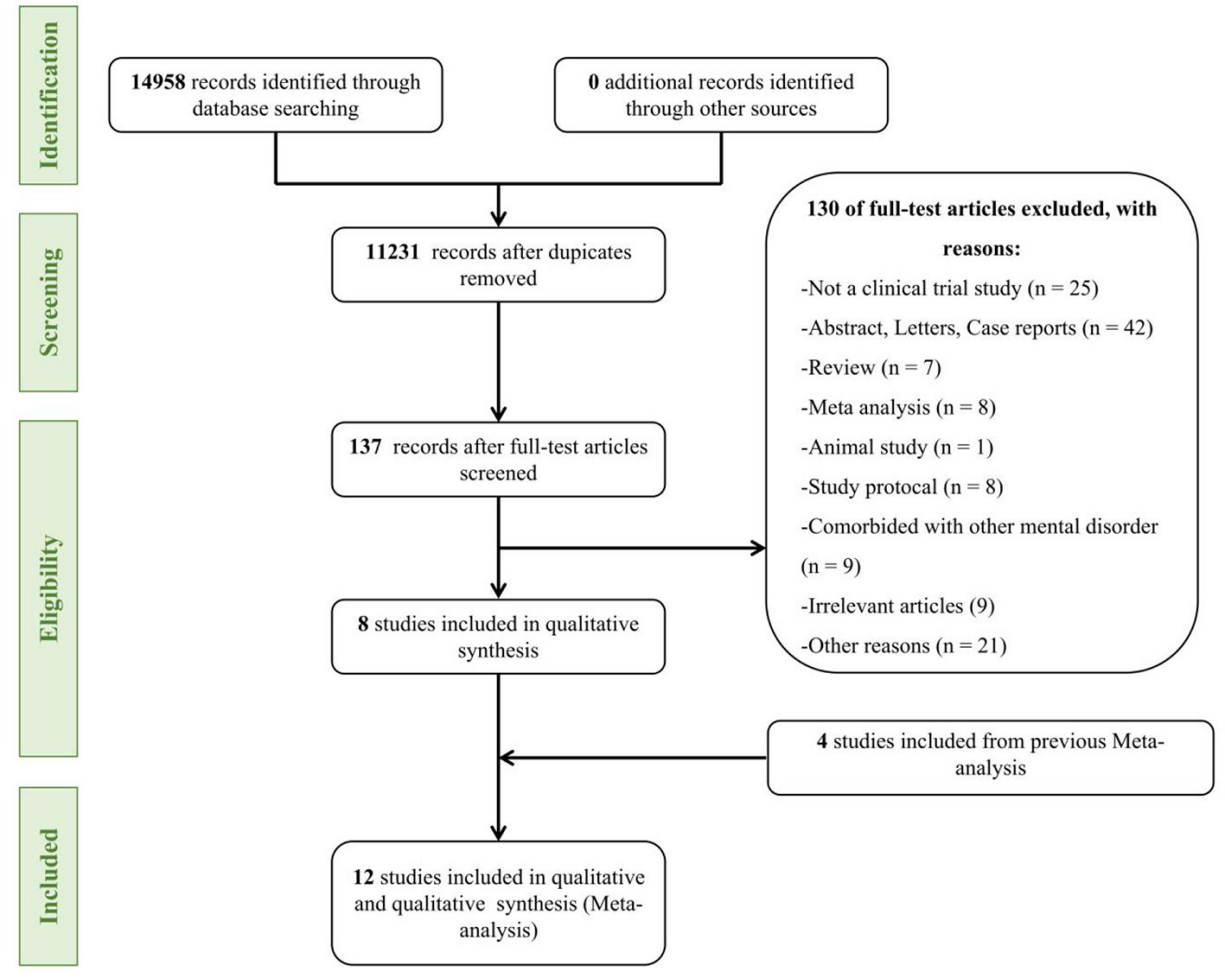
Flowchart of the study selection process.

Initial screenings identified 14,958 records from Web of Science, PubMed, Embase, and Cochrane Library databases. After removing duplicate publications, 11,231 articles were obtained. Forty-six systematic reviews and meta-analysis articles, 223 irrelevant article, and 1 animal research were excluded, 56 articles were obtained. In total, articles not meeting the selection criteria were excluded after reading the titles and abstracts, resulting in the exclusion of 270 articles. These records were screened, which led to full-text scrutiny of 56 articles. After carefully reading the 56 articles, 42 articles without a control group, four outcome indicators do not meet the inclusion criteria. Finally, 12 articles were included for meta-analysis, including a total of 371 subjects (Table 1). The process of literature screening is shown in Figure 1.

**Table 1:** Characteristics of randomized clinical trials included in the meta-analysis.

**References**	**Clinical characteristics**			**Depression characteristics**					**tDCS treatment**						**Cognitive performance**
	***N***	**% female**	**Age (mean** ±**SD)**	**Dx**	**TRD**	**Primary scale**	**Previous medication use**	**Treatment strategy**	**tDCS device**	**Anode**	**Cathode**	**Current, electrode size**	**Session duration (min/d)**	**Sessions**	**Test**
Bennabi et al. (2015)	24	64.4	60.1 ± 13.7	MDD	Y	HDRS-21	Stable doses	Augmentation	NeuroConn	F3	SO-R	2 mA; 35 cm^2^	30	10	TMT, MMSE, Isaacs Set Test, MIS, Picture naming test
Loo et al. (2010)	40	55	47.3 ± 12.2	MDD	N	MADRS	Stable doses	Add-on	NeuroConn	PF3	F8	1 mA; 35 cm^2^	20	5	RAVLT, TMT, Digit Span, COWAT, SDMT
Loo et al. (2012)	64	47	47.8 ± 12.5	MDD/ BD	N	MADRS	Stable doses	Add-on	NeuroConn	PF3	F8	2 mA; 35 cm^2^	20	15	RAVLT, Digit Span, Letter-number sequencing, COWAT, Stroop
Mayur et al. (2018)	16	37.5	44.9 ± 14.7	MDD	Y	MADRS	Not allowed	Augmentation	NeuroConn	F3	F4	2 mA; 25 cm^2^	30	10	MOCA, Visual memory score
Nord et al. (2019)	39	51.3	33.3 ± 10.6	MDD	Y	HDRS	Not allowed	Augmentation	NeuroConn	F3	Deltoid	1 mA; 35 cm^2^	20	8	back
Palm et al. (2012)	22	64	57 ± 12	MDD	Y	HDRS-24	Stable doses	Add-on	NeuroConn	F3	SO-R	1.5 mA; 35 cm^2^	20	10	Verbal Learning Memory Test, Regensburg Word Fluency Test, Letter Number Sequencing Task of the Wechsler Adult Intelligence Scale
Salehinejad et al. (2015)	30	56.6	28.3 ± 28.2	MDD	NI	HDRS	Not allowed	Monotherapy	TCT Research Limited	F3	F4	2 mA; 35 cm^2^	20	10	Delayed Matching to Sample, Pattern Recognition Memory
Salehinejad et al. (2017)	24	62.5	26.1 ± 5.8	MDD	NI	BDI	Not allowed	Monotherapy	Activa Dose Ionto- phoresis	F3	F4	2 mA; 35 cm^2^	20	10	Paired Associates Learning, Spatial Recognition Memory, Rapid Visual Information Processing; Choice Reaction Time
Moreno et al. (2020)	64	94	44.61 (11.82)	MDD	N	HDRS-17	Wash-out	Monotherapy	Soterix	F3	F4	2 mA;	30	22	MOCA, TMT, Digit span, SDMT, Verbal-Fluency, Processing Speed, Working Memory
Kumar et al. (2020)	18	72	66.33 (5.83)	MDD	N	MADRS	N/I	Add-on	Magstim^®^	F3/F4	Iz	1 mA; 25 cm^2^	30	10	N-back, Boston Naming Test, Brief Visuospatial Memory Test, Clock Drawing Test, Continuous Performance Test, California Verbal Learning Test, Digit Symbol Subtest, Stroop
Nejati et al. (2022)	20	100	30.35 ± 6.83	MDD	N	BDI	Wash-out	Add-on/ Monotherapy	ActivaTek Inc.	F3, Fp2	Fp2, F3	1.5 mA; 25 cm^2^	20	3	Go/No-Go, Stroop, 1-Back
Figeys et al. (2022)	10	40	77.10 ± 6.98	Depression, Anxiety	N	GDS, GDA	NI	NI	HDCStim	F3	supraorbital	1.5 mA; 35 cm^2^	20	10–14	SDMT, TMT, Digit span, Stroop

MDD, major depression disorder; BD, bipolar disorder; HDRS, Hamilton Depression Rating Scale; MADRS, Montgomery–Asberg Depression Rating Scale; BDI, Beck Depression Inventory; GDS, Geriatric Depression Scale; GDA, Geriatric Anxiety Inventory; TMT, Trail Making Test; RAVLT, Rey Auditory Visual Learning Test; COWAT, Controlled Oral Word Association Test; SDMT, Symbol Digit Modalities Test; MOCA, Montreal Cognitive Assessment; MMSE, Mini Mental State Examination; Y, yes; N, no; NI, not informed.

### 3.2 Basic characteristics and risk bias assessment of included literature

**Figure 2:**
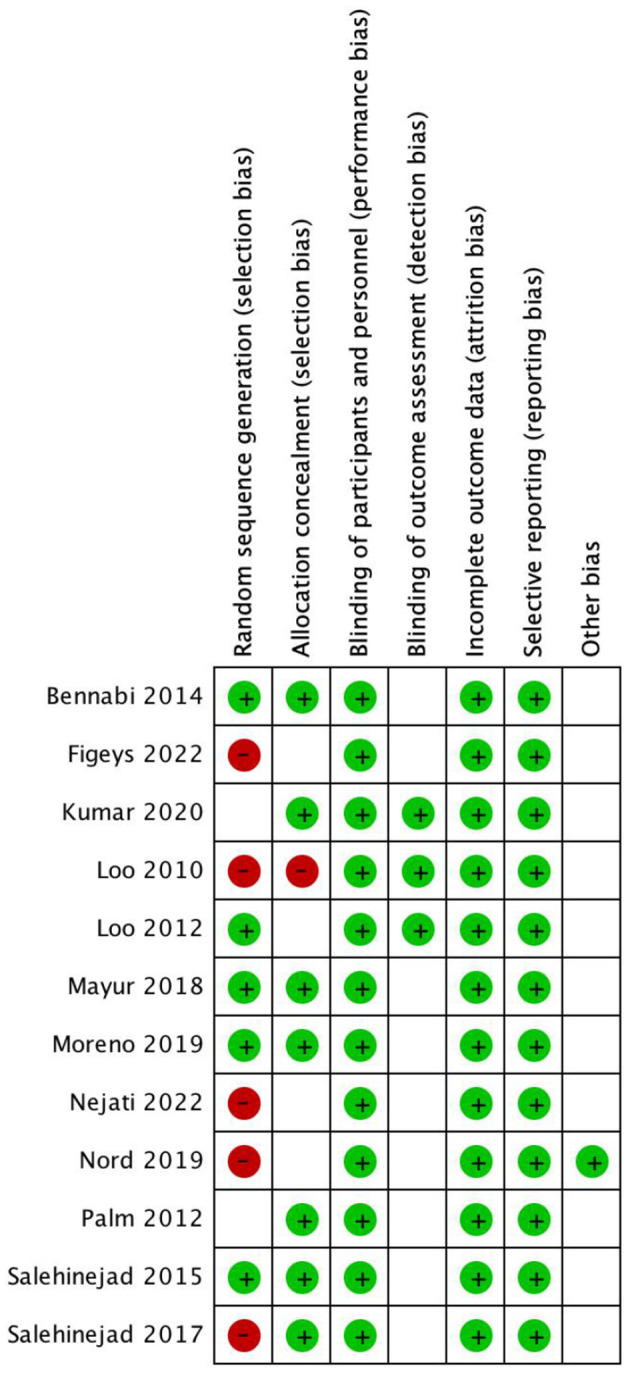
Funnel plots and tests of symmetry for publication bias.

The 12 articles finally included were all RCTs experiments. Due to the particularity of the intervention treatment, the design and implementation of random grouping may face certain challenges. All included articles reported attrition of patients, with relatively complete and test results, and no specific documentation describing concealment distribution. The basic characteristics of the literature are shown in Table 1, and the risk assessment of risk bias is shown in Figure 2.

### 3.3 The efficacy of antidepressant treatment of tDCS and its impact on cognition

#### 3.3.1 Overall effects of tDCS treatments for depression

**Figure 3:**
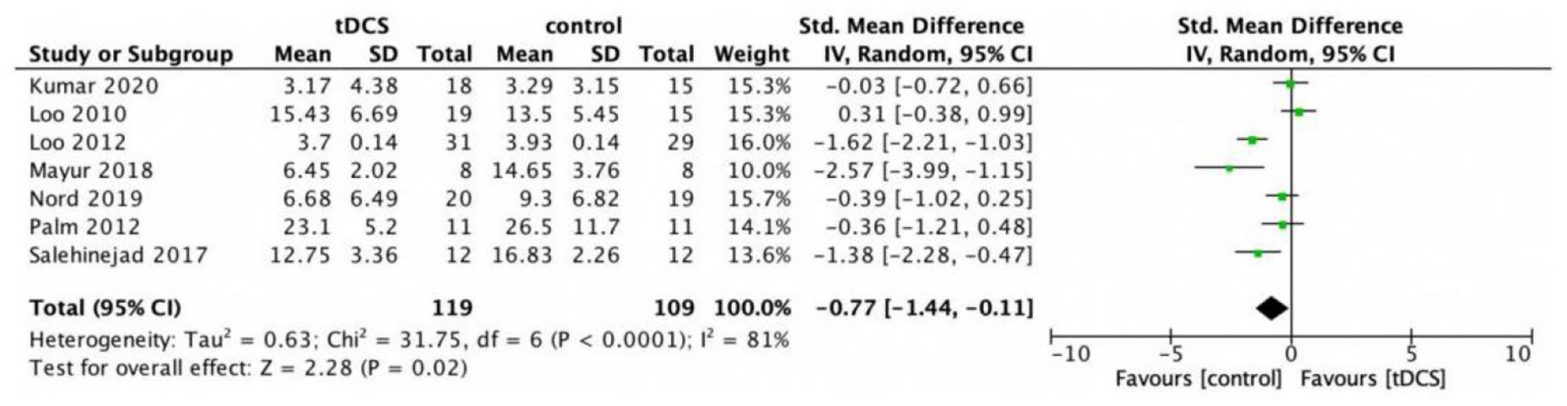
Forest plot of the efficacy of tDCS in the treatment of depression.

We evaluated the effectiveness of tDCS for improving depressive symptoms using endpoint depression scores, including a total of seven studies involving 191 patients. Meta-analysis results showed that active tDCS was more effective in antidepressant than in the sham group [*g* = −0.77, 95% CI (−1.44, −0.11); Figure 3].

#### 3.3.2 Results of global cognitive function and verbal memory measures

**Figure 4:**
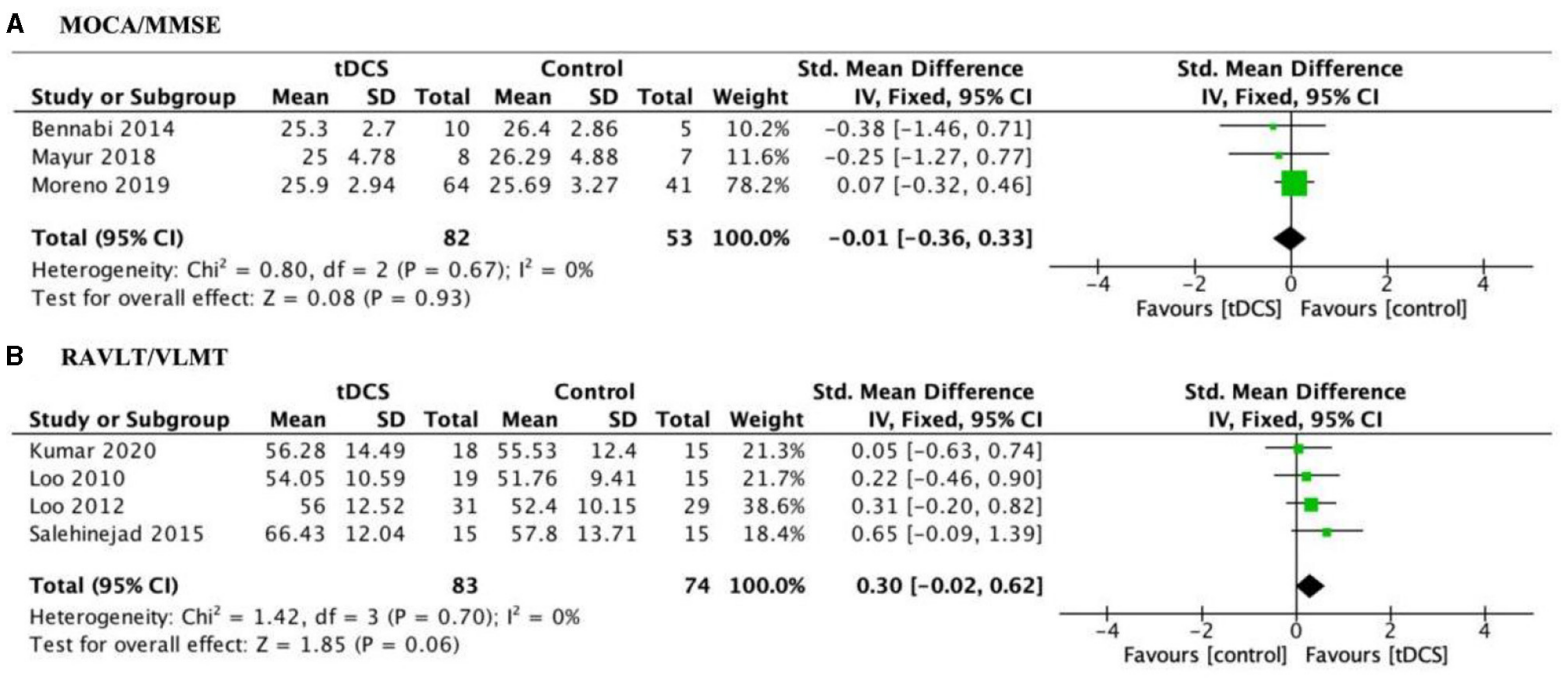
Forest plot of the impact of tDCS on global cognitive function **(A)** and verbal memory measures **(B)** in depression patients. MOCA, Montreal Cognitive Assessment; MMSE, Mini Mental State Examination; RAVLT, Rey Auditory Visual Learning Test; VLMT, Verbal Learning Memory Test.

Included studies used global cognitive function to assess cognitive improvements in tDCS treatment for depression patients. The pooled effect size at post-test across 3 tDCS studies was not significant, with *g* = −0.01, 95% CI (−0.36, 0.33); Figure 4A. Heterogeneity was low, *I*^2^ = 0%. In addition, we used RAVLT/VLMT to assess verbal memory performance for tDCS treatment with MDD patients. Finally, marginal significant effects were detected [*g* = 0.30, 95% CI (−0.02, 0.62); Figure 4B] when pooling four trials that specifically reported verbal memory deficits. This result indicates a trend toward improvement in verbal memory with active tDCS treatment compared to sham.

#### 3.3.3 Multi-aspects outcomes of cognitive function measures

**Figure 5:**
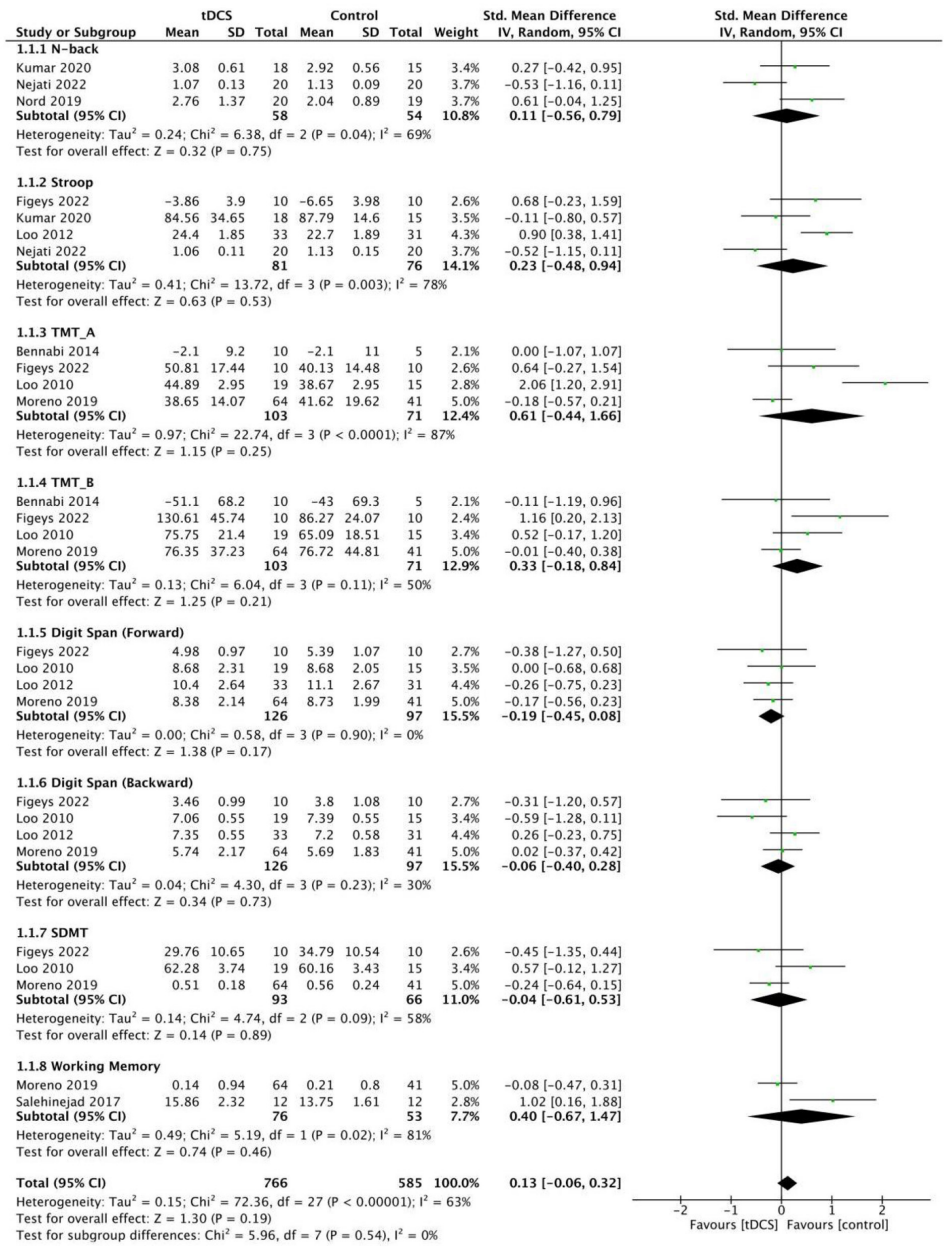
Cognitive function measures. TMT, Trail Making Test; SDMT, Symbol Digit Modalities Test.

Neuropsychological test, i.e., N-back, Stroop, and TMT-B was used to evaluate the effect of MDD patients' executive function. No significant effect sizes were observed in any of the examined specific outcomes: for N-back [*g* = 0.11, 95% CI (−0.56, 0.79); Figure 5], Stroop [*g* = 0.23, 95% CI (−0.48, 0.94); Figure 3], and TMT-B [*g* = 0.33, 95% CI (−0.18, 0.84); Figure 5].

Subgroup analysis of attention outcomes in MDD patients indicated that tDCS treatment was not better than that after sham group. Specifically, no significant effects were found when pooling 4 trials that consistently reported digit span, including forward [*g* = −0.19, 95% CI (−0.45, 0.08); Figure 5] and backward [*g* = −0.06, 95% CI (−0.40, 0.28); Figure 5]. And heterogeneity was low, with *I*^2^ = 0% and *I*^2^ = 30%. TMT-A was reported in four trials on patients with MDD. The pooled estimate demonstrated no significant effects at post-treatment [*g* = 0.61, 95% CI (−0.44, 1.66); Figure 5], whereas with high heterogeneity (*I*^2^ = 87%).

Three studies used neuropsychological evaluation to evaluate the efficacy of SDMT in MDD patients, totally 93 patients were included. Results found that there were no significant differences in SDMT between the two groups [*g* = −0.04, 95% CI (−0.61, 0.53); Figure 5]. At the same time, two studies used neuropsychological evaluation to evaluate the efficacy of working memory in MDD patients, totally including 76 patients. Results found that there were no significant differences in working memory between the two groups [*g* = 0.40, 95% CI (−0.67, 1.47); Figure 5]. Of note, N-back is the primary tool for assessing working memory capacity and attention control, whereas the other two working memory tests mentioned in the literature mainly evaluate short-term learning and memory. Thus, we analyzed these aspects distinctly in our final analysis.

### 3.4 Adverse events

**Figure 6:**
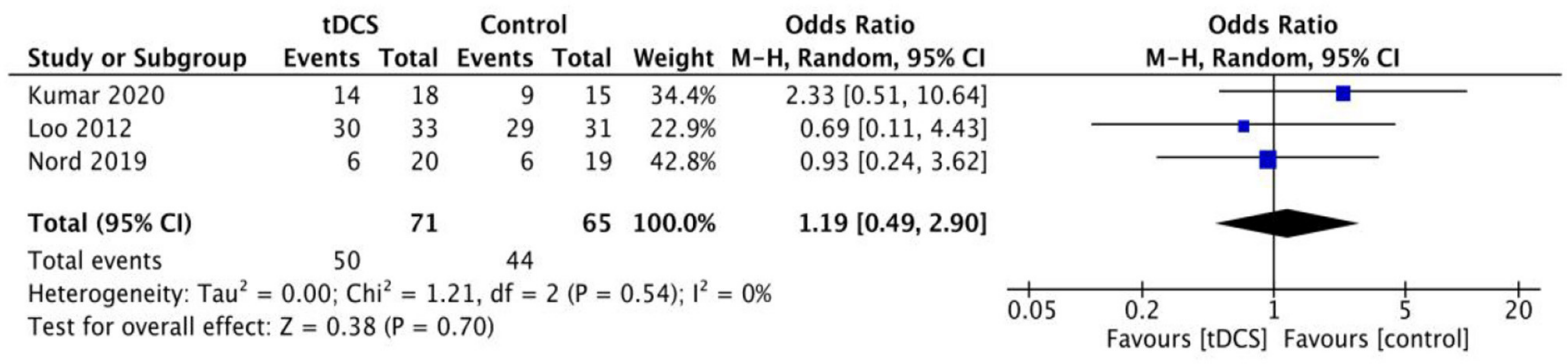
Adverse events.

All-cause adverse events were reported in 3 studies on pain, burning/heating sensation at anode and/or cathode site, headache etc. Pooling 3 trials that reported at least one adverse event of the conditions, we obtained a non-significant difference between the two groups [OR = 1.19, 95% CI (0.49, 2.90); Figure 6].

## 4 Discussion

This study presents a systematic review and meta-analysis of the clinical efficacy of tDCS in antidepressant treatment and its impact on cognitive function. It is worth noting that there were two studies on the current work topic (Martin et al., 2018), both of which provide valuable insights into the potential benefits of tDCS in enhancing cognitive function in patients with depression, and they are not without limitations. Firstly, the study by Martin et al., despite conducting a meta-analysis of individual patient data, had a relatively limited sample size and number of included studies, potentially affecting the generalizability and statistical power of the findings. Additionally, its examination of individualized differences and long-term effects was inadequate, limiting our understanding of response patterns across patient groups and the durability of treatment benefits. On the other hand, the systematic review by Jin et al., while comprehensive in scope, lacked a meta-analysis to provide quantitative effect estimates. This reliance on narrative descriptions limited the robustness of its conclusions. Furthermore, it lacked an in-depth exploration of the underlying mechanisms and offered only general suggestions for future research directions.

To address these limitations, by compiling data from twelve randomized, sham-controlled studies, we quantitatively analyzed cognitive performance across key cognitive domains, including global cognitive functioning, verbal memory, executive functioning, attention and working memory. For antidepressant efficacy, meta-analysis results showed that active tDCS was more effective than in the sham group for MDD patients, demonstrated that tDCS has a good antidepressant effect. However, our study did not demonstrate significant differences in the effects of global cognitive function for a treatment course of active tDCS compared to sham treatment in MDD patients, whereas there was a marginal significant difference in the effect of verbal memory, which underscores the potential for tDCS to enhance verbal memory in patients with MDD, and future studies with larger sample sizes or more targeted tDCS protocols may help clarify this effect. Additionally, the use of specific neuropsychological tests designed to detect subtle changes in verbal memory performance may aid in detecting statistically significant effects. Furthermore, we performed subgroup analyses to explore the effects of tDCS on eight cognitive function outcomes (e.g., attention, working memory). These analyses did not reveal any beneficial effects of active tDCS compared to sham treatment across the examined dimension-specific cognitive measures. In summary, our study provides an important evidence-based medical support for the antidepressant efficacy of tDCS and its dimension-specific cognitive benefits, contributing to a deeper understanding of its clinical applications.

Our study demonstrated that tDCS has a good antidepressant effect, which was consistent with the initial findings of Salehinejad et al. (2017) and Palm et al. (2012). Interestingly, this finding was driven largely by the Salehinejad et al. (2017) study where patients similarly received monotherapy treatment, who attributed the significant improvement in mood scores for depression to an ameliorate of patient's cognitive control deficits. However, this conclusion diverges slightly from the findings of Loo et al. (2018), who conducted a comprehensive international trial on tDCS for depression and demonstrated a null result. One potential explanation for this difference could be the relatively small sample size of our study. Additionally, based on our meta-analysis results, studies that followed a more standard daily or near-daily dosing schedule were included, and confounding variables such as drug dosage may have influenced the consistency of the findings across these studies. Given the ameliorating effect of tDCS on negative mood in MDD (Wang et al., 2022; Aust et al., 2022), electrical stimulation could be recognized as a complementary approach to maximize the therapeutic effect of psychological interventions on MDD.

We found no significant differences in the impact on global cognitive function between active tDCS and sham treatment courses. Notably, this result should be considered with caution, since the number of trials was small, and these analyses are likely underpowered. Our results align with a previous meta-analysis of seven randomized sham-controlled trials in adults with MDD, which also failed to show any cognitive benefits from tDCS (Martin et al., 2018). Indeed, some studies have shown positive effects of tDCS on cognition in patients with active MDD, these improvements may be attributed to mood enhancement (Gogler et al., 2017; Moreno et al., 2015; Wolkenstein and Plewnia, 2013). Nevertheless, our findings identified that patients who received active tDCS relative to those who received sham tDCS showed increased performance gains following the treatment course on the verbal memory measures. Studies in patients with cognitive disorders have shown the benefits of tDCS combined with cognitive tasks on visual memory, attention and executive function (Boggio et al., 2009; Nelson et al., 2014; Chen et al., 2022; Simko et al., 2021). The positive impact of this combination may arise from tDCS acutely enhancing attention or executive function during cognitive tasks, leading to improved learning outcomes (Kumar et al., 2020). Alternatively, tDCS treatment administered after cognitive tasks may contribute to memory consolidation (Vorobiova et al., 2019; Sandrini et al., 2019). Thus, future studies should explore both immediate and sustained effects of combining tDCS with cognitive stimulation to enhance global cognition in older patients with MDD (Oken, 2008; Fonteneau et al., 2019; Calamia et al., 2012).

Moreover, we performed subgroup analyses to investigate the impact of tDCS on eight cognitive function outcomes, and identified there was no beneficial effect of a treatment course of active tDCS compared to sham treatment across the examined dimension-specific cognitive measures. While a single tDCS treatment in patients with depression has been demonstrated to produce acute cognitive enhancement, our results indicated that there is no cumulative or long-term improvement effect when these courses are repeated. Additionally, the absence of differential cognitive effects may also be attributed to the possible lack of sensitivity of the tests that were included in these studies to detect subtle differences in cognition, particularly in attention states previously associated with tDCS (Gladwin et al., 2012). Another potential explanation could be the occurrence of type 2 error due to the small sample size.

Our analysis of tDCS revealed that it did not trigger any adverse side effects in patients with major depression, indicating its safety as an antidepressant treatment (Bares et al., 2019; Razza et al., 2023). Nonetheless, further research is needed, as some findings suggest enhanced practical gains in cognitive performance after tDCS. Additionally, we observed an enhancement in verbal memory in tDCS responders, and this finding also warrant confirmation in further studies. Future clinical trials employing tDCS should try to determine the optimal stimulus parameters, dose requirements, and predictors of a favorable treatment response, thereby enhancing its therapeutic potential.

As study strengths, this is the largest meta-analysis evaluating the efficacy of tDCS in depression to date. We implemented a rigorous assessment process for study eligibility and quality, ensuring that only high-standard research was included in our analysis. Furthermore, we utilized additional meta-analytical techniques to reinforce the robustness of our findings. Specifically, we conducted a thorough assessment of publication bias, which helped us to identify and account for any potential biases in the literature. Additionally, we employed both random-effects and fixed-effects models, providing a comprehensive and nuanced understanding of the data. These methodological strengths significantly enhance the credibility and reliability of our results, offering valuable insights into the potential of tDCS as a treatment for depression. Undoubtedly, our study also had some limitations. First, due to the limited studies on tDCS treatment for cognitive function in depressed patients, the sample size is relatively small. Therefore, although the meta-analysis was used to further expand the sample size in this study, it still needs to be enriched; Secondly, as the tDCS effects are shown to be more effective in individuals with more severe cognitive deficits, thereby, this confounding factor needs to be considered for future inclusion in the meta-analysis (Hill et al., 2016). Thirdly, we excluded RCTs that did not assess cognitive functions, potentially introducing bias into the sample. To address this limitation and provide a more comprehensive understanding of tDCS's effects on MDD, future studies should incorporate a wider range of RCTs, including those that investigate both the antidepressant and cognitive effects of tDCS. Additionally, larger-scale, well-designed RCTs are needed to validate our findings and further explore optimal treatment parameters, dosage requirements, and predictors of response to tDCS in MDD patients.

## Data Availability

The original contributions presented in the study are included in the article/supplementary material, further inquiries can be directed to the corresponding author.
